# Evidence-based treatment for treatment resistant schizophrenia

**DOI:** 10.1192/j.eurpsy.2026.10281

**Published:** 2026-07-31

**Authors:** P. Mohr

**Affiliations:** 1 National Institute of Mental Health, Klecany; 2 Third Faculty of Medicine, Charles University, Prague, Czech Republic

## Abstract

**Abstract:**

Treatment-resistant schizophrenia (TRS) is a common clinical problem, up to 30% of patients show little or no response to treatment. The most comprehensive definition of TRS was coined by the Treatment Response and Resistance in Psychosis (TRRIP) working group. First step in the management of TRS is to rule out pseudoresistance, i.e., failure to respond from different reasons (non-adherence, poor metabolizers, incorrect diagnosis, comorbidity, insufficient dosage or duration of trial, masking side effects). There is a general agreement among various treatment guidelines, the drug of choice for TRS is clozapine. Recent meta-analyses, systematic reviews, and individual meta-analyses cast doubts on clozapine superiority, suggesting that other antipsychotics (e.g., olanzapine, clozapine) may as effective as clozapine. However, it should be noted that in many RCTs clozapine was underdosed; numerous studies where clozapine arm was not blinded were excluded from analyses, severely resistant patients were not included, and finally, clozapine was significantly superior to comparator drugs when disregarding RCTs sponsored by the manufacturer of the comparator. Patients who do not respond or do not tolerate clozapine (“ultra-resistant schizophrenia”) may benefit from clozapine augmentation. Data indicate that clozapine can be effectively combined with ECT, mirtazapine, or memantine for overall symptoms, with ECT for positive symptoms, memantine, duloxetine, or mirtazapine for negative symptoms. Real-world data from Finnish and Swedish national registers showed that the dose of an augmenting agent is important (standard doses of SSRIs and SNRIs, medium doses of aripiprazole). Non-pharmacological therapeutic options for TRS, in monotherapy or as clozapine augmentation, include neurostimulatory methods (ECT, rTMS, tDCS), psychotherapy for specific symptoms (CBT), physical activity for negative symptoms, or more experimental (DBS, virtual reality).

**Disclosure of Interest:**

None Declared

